# Cultivation-independent high-quality microbial genome reconstruction from environmental samples with midi-metagenomics

**DOI:** 10.1101/gr.280099.124

**Published:** 2026-07

**Authors:** John Vollmers, Maximiano Correa Cassal, Anne-Kristin Kaster

**Affiliations:** 1 Institut für Biologische Grenzflächen 5, Karlsruhe Institute of Technology, 76344 Eggenstein-Leopoldshafen, Germany;; 2 Institute for Applied Biosciences, Karlsruhe Institute of Technology, 76131 Karlsruhe, Germany

## Abstract

Because the majority of microbial organisms still evade cultivation attempts, genomic insights into many taxa are limited to cultivation-independent approaches. However, current methods of metagenomics and single-cell genome sequencing have individual drawbacks, which can limit the quality and completeness of the reconstructed genomes. Current attempts to combine both approaches still use whole-genome amplification techniques, which are prone to bias. Here, we propose a novel approach for the purpose of genome reconstructions that utilizes the potential of cell sorting for targeted enrichment and depletion of different cell types to create distinct cell fractions with sufficient DNA amounts, circumventing amplification. By distributing sequencing efforts over these fractions as well as the original sample, coassemblies become highly optimized for coabundance variation–based binning approaches. “Midi-metagenomics” enables accurate metagenome-assembled genome (MAG) reconstruction from individual sorted samples with higher quality than coassembly and binning of multiple distinct samples and therefore improves analyses of uncultivated microorganisms.

The vast majority of prokaryotes still evade cultivation attempts under laboratory conditions and therefore cannot be subjected to direct analysis via classic culture-based microbiological and biochemical methods (Steen et al. 2019; Bodor et al. 2020). The discrepancy between the small number of cultured prokaryotic strains compared with the vast diversity and ubiquity of uncultured microbes, commonly referred to as “microbial dark matter” (MDM) (Marcy et al. 2007; Rinke et al. 2013; Solden et al. 2016; Zha et al. 2022), represents a large reservoir of biotechnological and/or pharmaceutical potential that is still untapped (Bodor et al. 2020; Kaster and Sobol 2020; Escudeiro et al. 2022).

Advances in cultivation-independent methodologies, such as metagenomics and single-cell genomics (SCGs), have enabled sequence-based predictions of phylogenetic and functional characteristics of uncultivated microorganisms (Fig. 1; Tyson et al. 2004; Solden et al. 2016; Vollmers et al. 2017), but with each method having distinct advantages and disadvantages. In metagenomics (Fig. 1A), bulk DNA from a mixed community, such as an environmental microbiome, is extracted and sequenced (Schmeisser et al. 2007). Subsequent analyses steps can then reveal the phylogenetic and functional diversity of a given community and even enable the reconstruction of so-called “metagenome-assembled genomes” (MAGs) from uncultivated individual community members via contig-based binning methods (Liu et al. 2025). Binning of metagenomic contigs is a challenging and error-prone process, especially for highly complex communities (Ma et al. 2023) and low abundant organisms (Qayyum et al. 2025). As a result, MAGs are highly susceptible to chimerism and can show varying degrees of fragmentation and completeness in addition to contamination (Orakov et al. 2021; Vollmers et al. 2022). Furthermore, MAGs are often limited to the most abundant species present in the sample and may not reliably resolve strain variants or elements of horizontal gene transfer (Vollmers et al. 2017).

**Figure 1.**
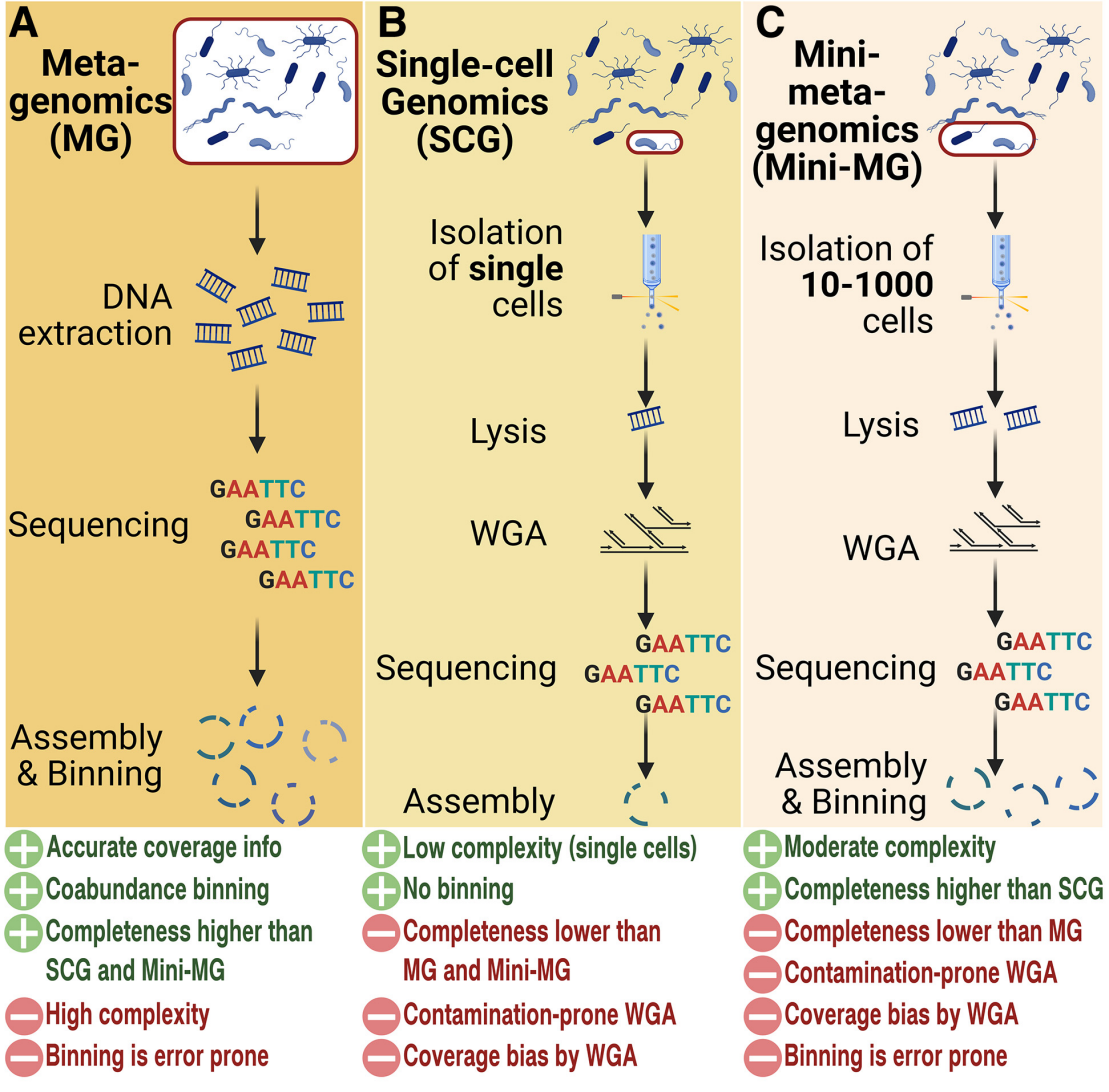
Current culture-independent methodologies. (*A*) In metagenomics, the entire DNA of an environmental community is sequenced. Assembled contigs are binned into metagenome-assembled genomes (MAGs). (*B*) In single-cell genomics (SCGs), individual cells are isolated, sequenced, and analyzed. Because of little DNA content per cell, whole-genome amplification (WGA) is required. (*C*) In mini-metagenomics, typically pools of five to 1000 cells are sortied and sequenced. Although complexity is lower than for standard metagenomics, binning is still required, and the low DNA content of small cell pools still necessitates WGA. Created with BioRender (https://www.biorender.com).

SCGs (Fig. 1B) circumvent this problem by directly targeting individual cells, thereby enabling reliable genome resolution on strain level (Xu and Zhao 2018; Kaster and Sobol 2020). However, because a single prokaryotic cell contains only a few femtograms of DNA, a whole-genome amplification (WGA) is required because the minimum requirement for high-throughput sequencing is typically in the nanogram range (Hutchison and Venter 2006; Kalisky and Quake 2011). This is a severe disadvantage, as WGA not only is expensive and prone to contamination but usually yields extremely uneven read coverage, constituting bias that is particularly pronounced for genomes with high GC content and usually results in single-cell amplified genomes (SAGs) that are typically even more fragmented and incomplete than MAGs (Lasken and Stockwell 2007; Alteio et al. 2020; Kaster and Sobol 2020).

To minimize these drawbacks and maximize the advantages of both methods, there is a strong interest in combining single-cell and metagenomic approaches. A current example for such an attempt is “mini-metagenomics” (Yu et al. 2017), which targets small groups of 10–1000 cells (Fig. 1C). These cells are then sequenced together and subsequently treated as a simplified metagenome to efficiently reduce random amplification bias (Yu et al. 2017; Alteio et al. 2020). The DNA yield of such a small amount of cells is, however, still not sufficient to circumvent amplification. The relatively low complexity of such mini-metagenomes should, in theory, allow for better genome reconstructions than the more complex metagenome of the original community; however, this approach is still affected by systematic WGA bias, which may be caused by, for example, variations in GC content (Marine et al. 2014). Most importantly though, effective binning criteria are limited because contig abundance information is not available owing to the uneven read coverage, a severe drawback that also obstructs the currently most effective binning strategy: coabundance variation across samples (Alneberg et al. 2014; Mattock and Watson 2023). Therefore contigs will likely have to be binned exclusively based on nucleotide signatures, which is less reliable, especially for short contigs of highly fragmented genomes (Vollmers et al. 2022; Mattock and Watson 2023).

We here present an alternative approach, termed “midi-metagenomics,” that utilizes cell sorting to create custom community fractions of sufficient cell count to circumvent the need for amplification entirely. Fluorescence-activated cell sorting (FACS) is used for targeted enrichment and depletion of different cell types to create fractions that are highly optimized for coabundance variation–based binning approaches. This way, the quality of genome reconstructions can be maximized, even if only individual samples without spatial or temporal parallels are available.

## Results

### Established workflow

**Figure 2.**
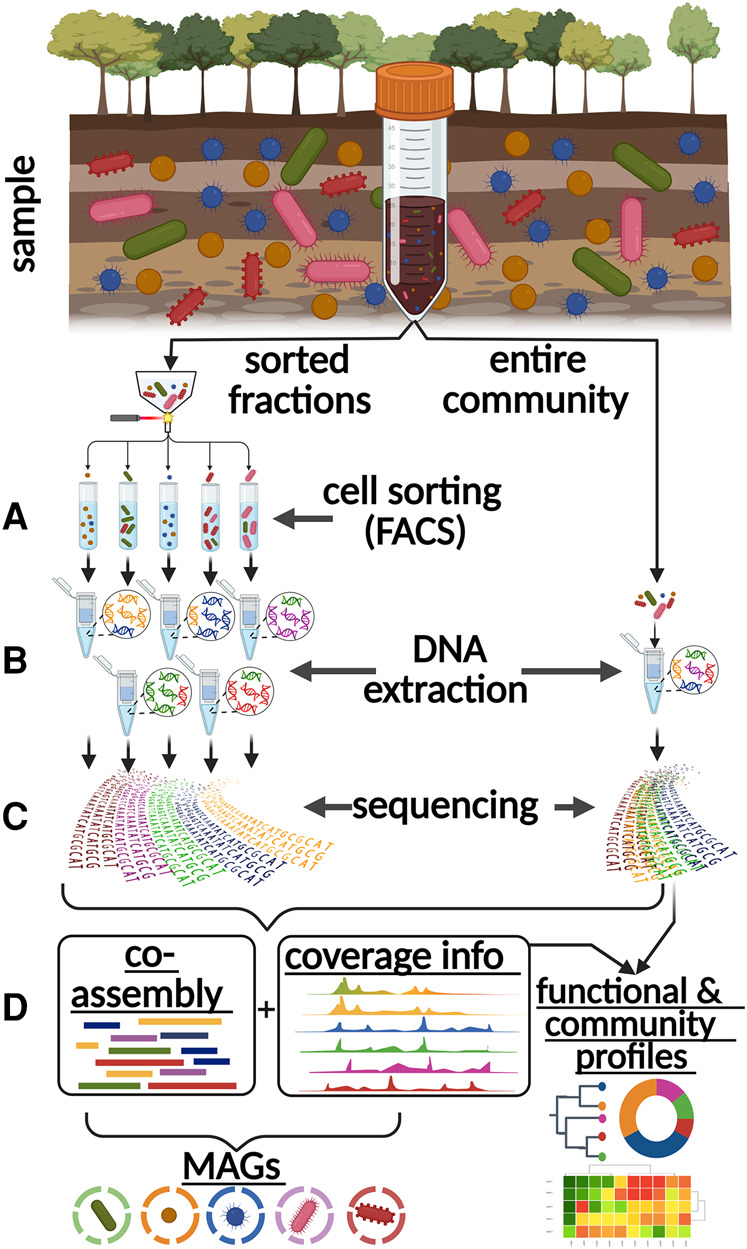
Midi-metagenomics workflow. (*A*) Part of the sample community is fractionated into distinct groups of several hundred thousand to millions of cells by cell sorting. (*B*) Different cell types are not separated with absolute stringency, but differentially enriched DNA is extracted separately from each fraction, as well as the original unsorted sample. (*C*) Extracted DNA is sequenced directly without whole-genome amplification (WGA). (*D*) Because the resulting read data sets represent different enrichments based on the same original community, they are optimal for coassembly as well as coabundance variation–based binning approaches. An unbiased representation of the source community is achieved by also including the original unsorted sample in the analyses. Created with BioRender (https://www.biorender.com).

In midi-metagenomics, the original sample population is divided into multiple fractions, in which different community members are selectively enriched or depleted via FACS (Fig. 2A). Possible strategies for selectively fractionating a complex community into distinct subpopulations are manifold (Woyke et al. 2017; Sturm et al. 2023). However, in this proof-of-principle study, sorting was based on relatively simple gating strategies exploiting only cell characteristics easily detectable via FACS: cell size as determined by forward scatter and “complexity” representing cell structures and granularity as defined by side scatter gating (Supplemental Fig. S1; Lavigne et al. 1997). Because soil represents one the most complex and challenging microbial communities for metagenomic analyses (Vollmers et al. 2017; Jansson and Hofmockel 2018), it was chosen as a test environment instead of controlled artificial consortia, which may not faithfully represent the diversity and dynamics of natural microbiomes.

In contrast to standard single-cell and “mini-metagenomic” approaches, which require an amplification step (Rinke et al. 2013; Yu et al. 2017; Alteio et al. 2020), the midi-metagenomic methodology utilizes bulk sorts of several hundred thousand to millions of cells into the same fraction. Preliminary trial DNA extractions performed on bulk sorts of bacterial cultures and soil samples indicated the presence of DNA predominantly in the supernatant and not the pellet (Wiegand et al. 2021) of centrifuged cell suspensions after FACS, especially in the case of soil samples (Supplemental Fig. S2). This observation indicates possible cell damage caused by the sorting process and subsequent release of cellular DNA (Mollet et al. 2008; Blainey 2013; Binek et al. 2019; Wiegand et al. 2021). Therefore, an adapted DNA extraction protocol was used for midi-metagenomic fractions, which includes an alcohol precipitation step directly from sorted cell suspensions rather than centrifuged cell pellets, thereby ensuring maximized DNA yields (Fig. 2B). In preliminary trial runs, DNA yields ranged between 5 and 30 ng DNA for up to 5 million sorted cells (Supplemental Table S1), which is more than sufficient for direct sequencing (Sato et al. 2019; Ribarska et al. 2022). Therefore, based on the low input requirements of <1 ng (Parkinson et al. 2012; Bowman et al. 2013; Tvedte et al. 2021) for modern sequencing library preparation techniques, sorting efforts may be reduced to 10,000–1,00,000 cells per sorted fraction in the future. The separate sequencing of genomic DNA for each fraction, as well as the original unfractionated sample (Fig. 2C), resulted in multiple distinct data sets for each sample.

#### Efficiency of cell sorting–based community fractionation

**Figure 3.**
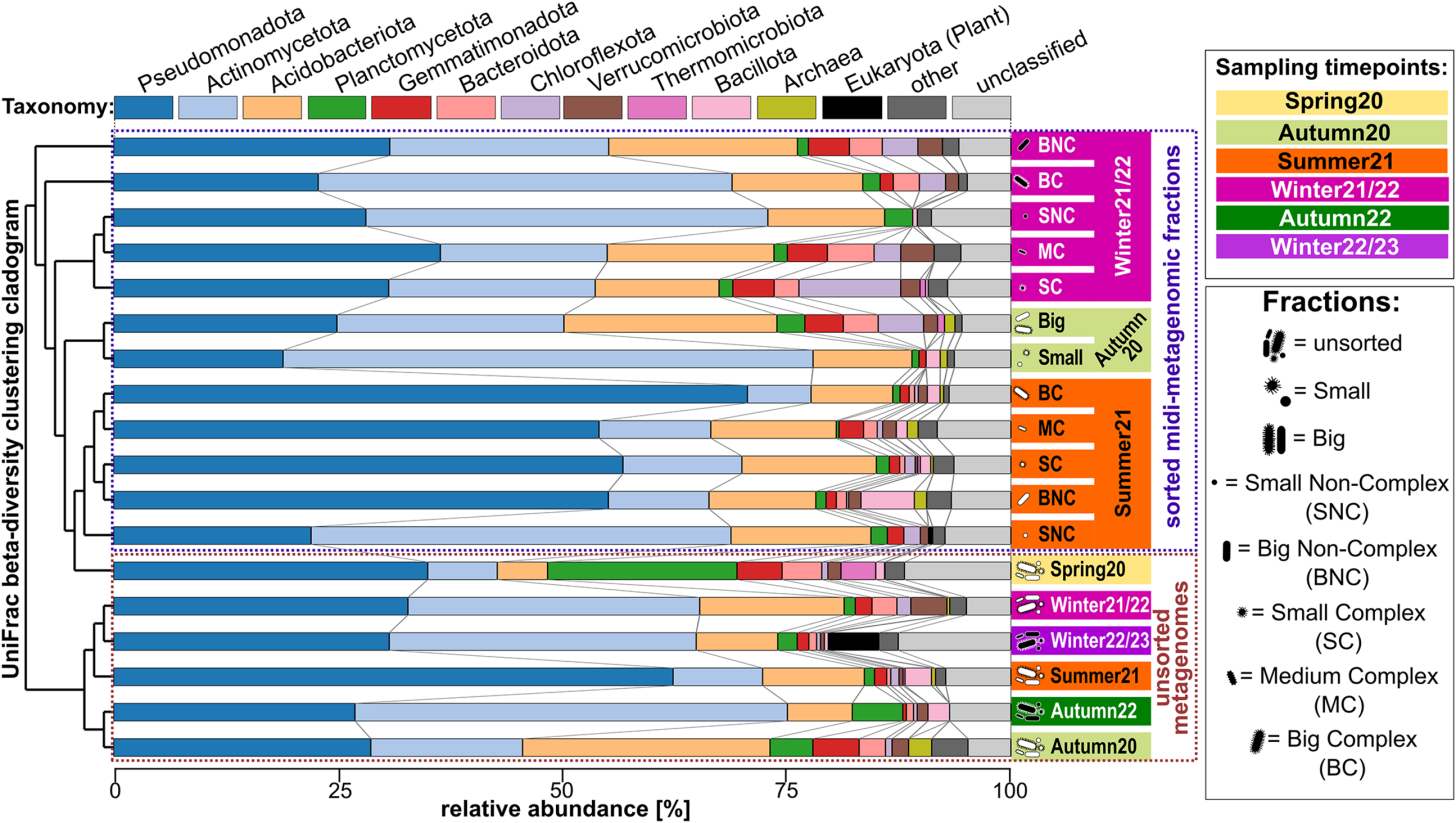
16S rRNA gene-based diversity among different (midi-)metagenomic fractions of different samples. Clustering is based on weighted UniFrac (Lozupone et al. 2011) beta-diversity scores and is shown as a cladogram on the *left*. The background coloring of *y*-axis labels on the *right* side indicates the respective origin-sample. Stacked bar charts indicate the community composition of each sample and fraction, with different phyla being indicated by a distinct color code as indicated *above* the plot, and relative abundances being indicated by bar heights according to the *x*-axis *below* the plot. Sorted midi-metagenomic fractions are indicated by pictograms, and abbreviations are given in the legend on the *right*.

The relationship between sorted fractions and corresponding unsorted samples was analyzed based on 16S rRNA gene diversity within the assembled metagenomic and midi-metagenomic fractions (Fig. 3; Supplemental Table S2) as well as 16S rRNA amplicons with increased sequencing depth (Supplemental Fig. S3; Supplemental Table S3). Weighted UniFrac scores calculated from these analyses show higher beta-diversities between sorted fractions and their respective nonfractionated communities than between nonfractionated samples taken at different time points (Fig. 3). This increased beta-diversity represents a strong shift in relative taxon abundances within the respective microbial communities, which can be exploited for distinguishing different organisms based on differential coverage information during downstream binning attempts.

At the same time, all sorted fractions show decreased alpha-diversity values and, therefore, lower community complexity compared with their respective nonfractionated counterparts (Supplemental Fig. S4). One sample exclusively sorted on forward scatter signals (which roughly indicate cell size) clusters closer to the unsorted sample when analyzed on high-depth amplicon sequencing level (Supplemental Fig. S3). This indicates that the use of the forward scatter alone provides a less systematic separation of the community compared with sorting based on combined forward and side scatter, possibly owing to the reduced resolution of morphological differences. Additional sorting metrics such as (auto)fluorescence should therefore even further improve binning efficiency.

#### Assembly and binning performance

**Figure 4.**
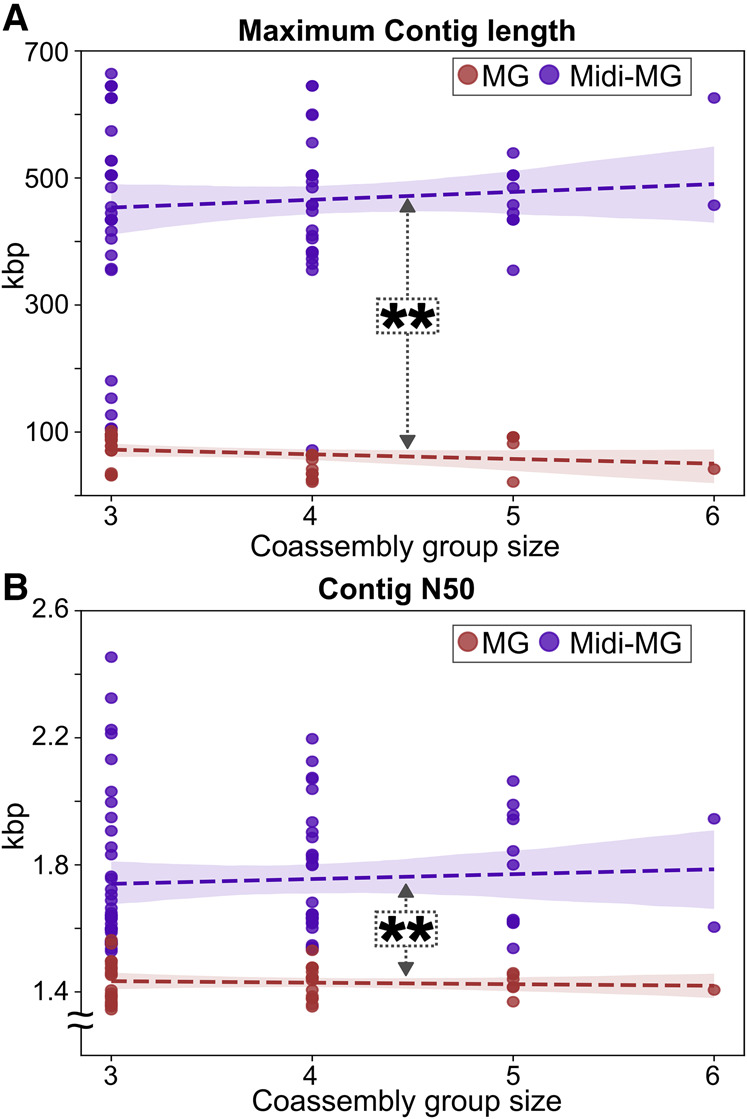
Comparison of assembly metrics for metagenomic and midi-metagenomic approaches in dependance on coassembled samples and fractions. Scatterplots showing metagenomic results as red, and midi-metagenomic results as purple dots. Trendlines and corresponding confidence areas were determined by regression analysis and are indicated by dashed lines and background coloring, respectively. (*A*) Maximum contig lengths. (*B*) Contig N50 values. The significance of differences in the distribution between metagenomic and midi-metagenomic assembly metrics were determined via Mann–Whitney *U* tests (MacFarland and Yates 2016). (MG) Metagenome, (Midi-MG) midi-metagenome.

Coassemblies of standard bulk metagenomics and midi-metagenomics were compared using the same total sequencing depth of 15 Gbp (averaging at 70 million read pairs per coassembly), equally distributed across the combined samples and fractions (Supplemental Table S4). Based on maximum contig length and N50 metrics, midi-metagenomic coassemblies of sorted fractions originating from the same original sample were significantly less fragmented than were coassemblies of distinct bulk metagenomic samples (Fig. 4), with *P* < 0.001 as determined via Mann–Whitney *U* tests (MacFarland and Yates 2016).

**Figure 5.**
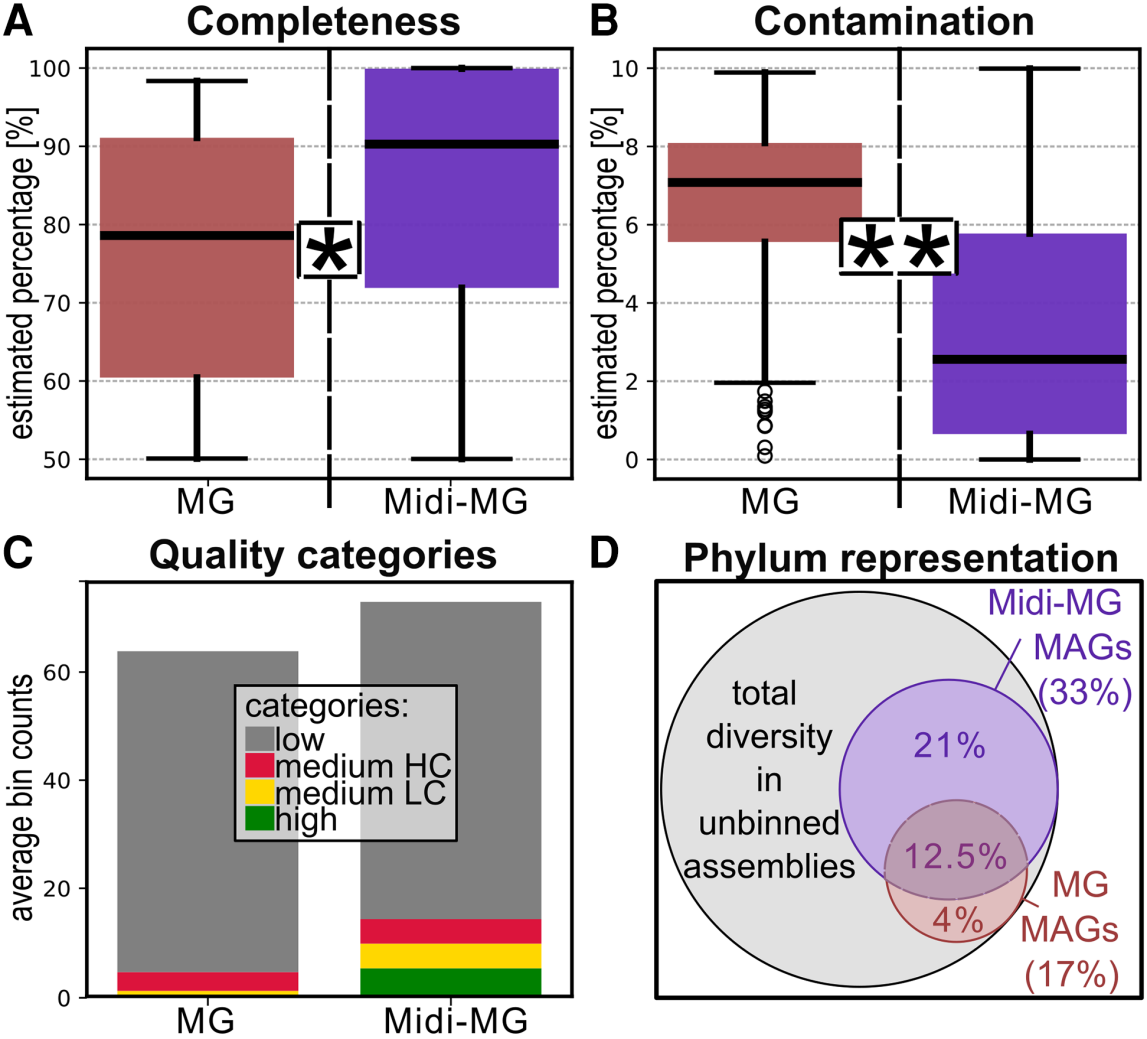
Comparison of quality metrics and diversity of MAGs obtained from standard metagenomic and midi-metagenomic coassemblies. (*A*,*B*) Boxplots showing the distribution of CheckM2 completeness and contamination estimates, respectively. The difference between metagenomic and midi-metagenomic results was found to be statistically significant (*P* < 0.01 based on the Moods median test) (Gibbons 2005) in both cases. A more detailed plot showing individual results is given in Supplemental Figure S5. (*C*) Average number of MAGs belonging to different quality categories obtained by standard metagenomics and midi-metagenomics. (*D*) Relative fractions of total phylum level diversity detected in the unbinned metagenomic coassemblies that are represented by metagenomic and midi-metagenomic MAGs, respectively. (MG) Metagenome, (Midi-MG) midi-metagenome.

Improved assembly metrics also affect the distribution of quality categories among the produced MAGs, as defined by the “minimum information about a metagenome-assembled genome” (MIMAG) standard, developed by the Genomic Standards Consortium (Bowers et al. 2017): Even before contamination filtering with MDMcleaner (Vollmers et al. 2022), midi-metagenomic approaches produced far more high-quality MAGs (completeness >90%, contamination <5%) compared with standard metagenomic assemblies, which predominantly consisted of only moderate-quality genomes (completeness >50%, contamination <10%) or low-quality genomes (either completeness <50% or contamination >10%) with contamination values typically >5% (Fig. 5A–C; Supplemental Tables S5, S7). These trends persist across different midi-metagenomic samples and coassembly subset groups of different sizes, despite varying community complexities, proving the robustness of the approach.

**Figure 6.**
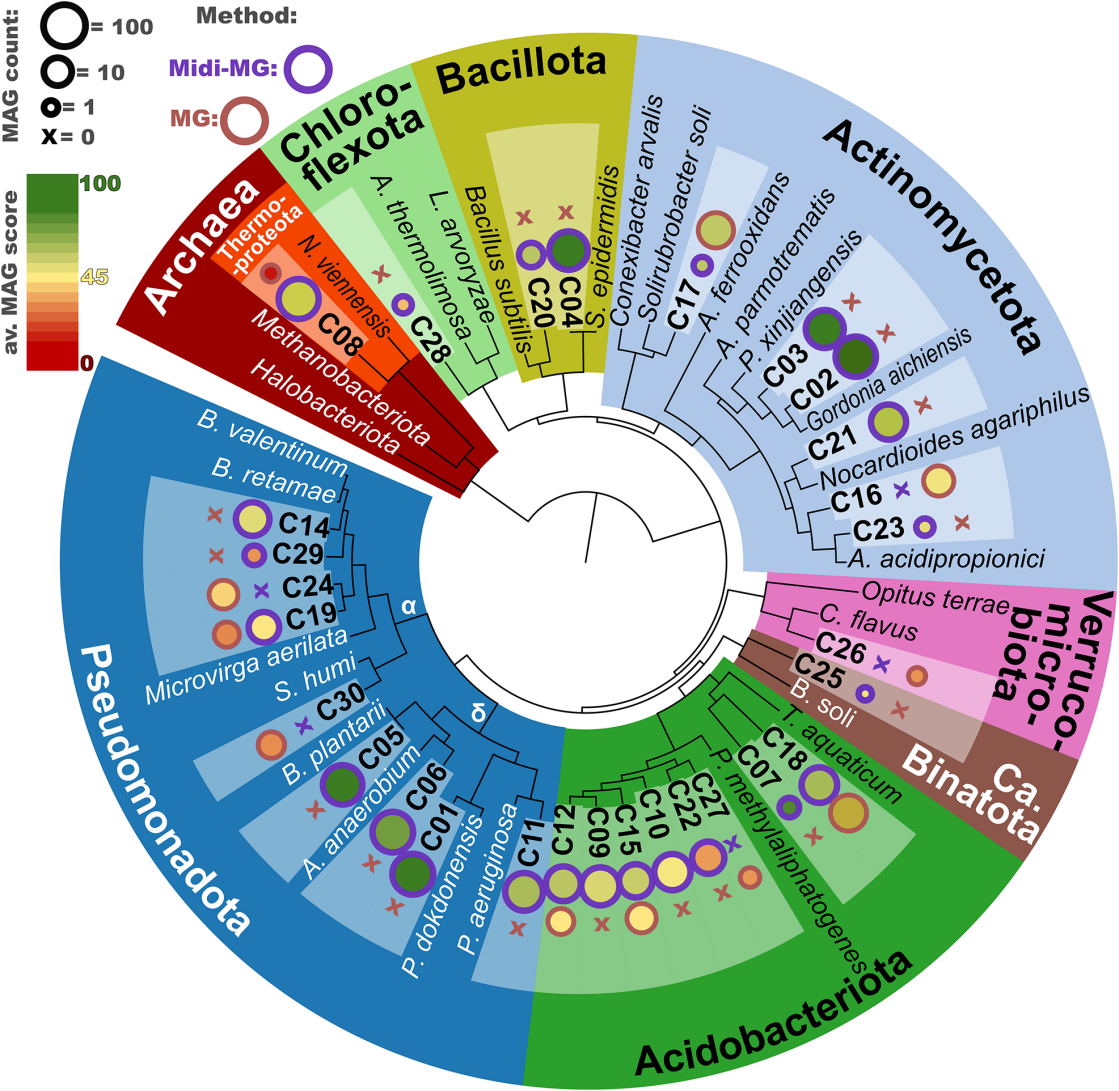
Multilocus sequence analysis (MLSA)–based phylogeny of representative MAGs and related reference genomes. Maximum likelihood phylogenetic clustering based on 61 single-copy orthologs shared by all comparison genomes, concatenated to a total length of 7178 amino acids. The software tool dRep (Olm et al. 2017) was used to group all MAGs on species level based on ANI comparisons and to rank the members of each group based on genome quality. Only groups with representatives showing >50% completeness and ≤5% contamination were considered, resulting in 30 groups labeled C1–C30, for each of which only the best representative is compared. Bubble plots next to each group designation indicate the number and average dRep score of each group, as indicated by the legend on the *upper left*. (α) Alphaproteobacteria, (δ) gammaproteobacterial, (MG) metagenomics, (Midi-MG) midi-metagenomics.

Interestingly, midi-metagenomic MAGs represented almost twice as many distinct phyla compared with standard MAGs (Fig. 5D), illustrating another important aspect of improved assembly and binning metrics, namely improved representation of original sample diversity. This is further corroborated by detailed phylogenomic analyses of low-contaminated MAGs (<5% contamination estimate) with at least moderate (50%) completeness (Fig. 6), which indicate a far broader and, owing to increased MAG qualities, also more reliable phylogenomic representation by midi-metagenomic MAGs compared with standard metagenomics. In addition, in the midi-metagenomic approach, a higher diversity of closely related but still distinct genomes could be reconstructed (as shown in Fig. 6 in the case of Acidobacteriota, and to a lesser extent also for Alphaproteobacteria and Actinomycetota), indicating a better resolution of sequence homologies between closely related organisms with this new method.

### Comparison with alternative assembly and coverage distribution strategies

Although the coassembly of different fractions or samples enhances read coverage for assembly and coabundance based binning, there is some debate about its applicability for standard metagenomics, as seasonal or regional variations may introduce complexities that may obstruct optimal assembly (Olm et al. 2017). To present an objective comparison of the performance of midi-metagenomics versus metagenomics, we therefore tested alternative assembly strategies for the same overall sequencing depth of 15 Gbp (Supplemental Fig. S6). A common strategy is to perform separate single assemblies for each sample and then map every read data set against each individual assembly (Olm et al. 2017). This strategy greatly reduced the performance of both metagenomic and midi-metagenomic approaches, likely owing to the reduced read depth negatively affecting assembly, thereby causing an increase of “low-quality” MAGs and reducing the yield of “moderate-quality” to “high-quality” MAGs (Supplemental Fig. S6A). Nonetheless, midi-metagenomics still performed at least as well as standard metagenomics under these conditions.

Another potential alternative sequence coverage distribution strategy is to focus sequencing and assembly efforts only on one “main” sample and to supplement with additional lower depth “auxiliary” read data sets meant exclusively for mapping and binning purposes. However, in our trials, this strategy could not mitigate the negative effects of the “single assembly” strategy for standard metagenomics, instead only showing a slightly positive effect for midi-metagenomic data sets, which thereby outperformed standard metagenomics also under these conditions (Supplemental Fig. S6B).

#### Comparison with mini-metagenomics

For comparison purposes, mini-metagenomics was also applied to one sample. This approach is designed to reduce MDA bias by supplying higher amounts of input DNA by sorting and lysing multiple cells (Fig. 1C). Accordingly, we encountered fewer negative MDA reactions and more complete genomes using this approach compared with standard SCGs. However, only two moderate-quality mini-metagenomic MAGs could be recovered, both displaying high contamination estimates close to the MIMAG cutoff of 10% (Supplemental Table S5; Supplemental Fig. S6C).

## Discussion

Midi-metagenomics integrates cell sorting and metagenome sequencing approaches into a new workflow that is optimized for high-quality MAG reconstruction. The key step of this approach is the seperation of the sampled microbiome into highly comparable but nevertheless distinct fractions of reduced complexity (Fig. 3; Supplemental Fig. S2) that can be sequenced directly without involving WGA methods (Fig. 2).

Because the resulting fractions represent subsets of the same community at the exact same time point, they are optimally suited for a coassembly strategy, maximizing the available sequencing depth for assembly as well as binning purposes. Accordingly, midi-metagenomics yielded significantly better coassembly metrics and MAG qualities compared with standard metagenomics (Figs. 4, 5), the latter having produced significantly shorter contigs and lower-quality MAGs, likely owing to intersample heterogenities, which are known to generally affect coassemblies of temporally or spatially distinct samples (Olm et al. 2017). The common alternative strategy of separately assembling each sample for metagenomics did also not improve assembly and binning outcomes (Supplemental Fig. S6), because it severely limits the read depth available for assembly per sample (Hofmeyr et al. 2020). Compensating this effect requires substantially increased sequencing efforts and costs to reach optimal coverage for each individual sample (Ma et al. 2023; Supplemental Table S7). This limitation is especially critical given that coabundance-based binning performs best across large numbers of parallel samples (Alneberg et al. 2014; Han et al. 2025), theoretically requiring deep sequencing of each individual sample.

Systematic comparisons with multiple independently published metagenome studies also confirm a generally lower efficiency of MAG reconstruction for metagenome approaches compared with midi-metagenomics, which yielded 411 high-quality MAGs across all midi-metagenomic samples, with an average of three per coassembly subgroup (Supplemental Table S7). A large-scale analysis by Ma et al. (2023) of several thousands of publicly available as well as newly generated soil metagenomes established a direct correlation between sequencing depth and the number and quality of generated MAGs. The sequencing depths in that study ranged from 0.03 to 146 Gbp, with an average depth of 14 Gbp (Supplemental Table S7), which is comparable to the soil metagenome control used in our study. As to be expected, the number of high-quality genomes produced per metagenome varied drastically from zero to 72, averaging at three, with larger numbers being generated at extreme sequencing depths of ≥100 Gbp. Interestingly, ∼41% of the 3304 data sets in that study did not yield any high-quality MAGs, indicating a generally relatively low efficiency of standard metagenomic approaches. Similar results were observed for two independent metagenome studies of agricultural soil samples (Supplemental Table S7; Nelkner et al. 2019; Hu et al. 2025), analyzing eight to 24 samples at depths of 10–12 Gbp each. These two studies yielded considerably lower counts of high-quality MAGs compared with midi-metagenomics despite surpassing the overall sequencing effort three- to 16-fold. These comparisons also showcase that a large fraction of metagenome samples are restricted to moderate- or lower-quality MAGs, mostly likely owing to interspecies homologies affecting assembly and binning. In contrast, midi-metagenomics yielded high-quality MAGs in almost every combination of samples and fractions and in larger proportions than any of the aforementioned approaches, indicating a far more efficient distribution of sequencing and sampling efforts. The only study to reach an equivalent quality of MAGs for soil samples via standard metagenomics was a comparison of metagenomics and mini-metagenomics by Alteio et al. (2020), which compared the genome reconstructions obtained from four deep sequenced soil metagenomes and 359 mini-metagenomes. This study, however, used a considerably higher overall sequencing depth of ∼200 Gbp, averaging at ∼50 Gb per sample, illustrating that standard metagenomics require extreme sequencing depths to guarantee high-quality genome reconstructions from soil samples, which is not financially feasible in most cases.

Our comparisons also indicate a higher effectiveness (Supplemental Fig. S6) as well as cost efficiency of the midi-metagenomic approach compared with mini-metagenomics, which showed at least nine times higher sequencing costs per MAG (Supplemental Table S6). A major issue with mini-metagenomics is that it targets multiple cells but still relies on WGA, which introduces bias and makes coabundance variation–based binning infeasible, thereby foregoing the main advantages of both SCGs and metagenomics. These conclusions are corroborated by the results of a more thorough mini-metagenomic sampling effort by Alteio et al. (2020), mentioned above. Although the mini-metagenomic MAGs obtained during that study did show lower contamination values than those of standard metagenomics, they also showcased a low overall efficiency of the approach, with few high-quality MAGs being obtained from only 0.8% of the samples, despite a high combined sequencing effort of >190 Gbp (Supplemental Table S7).

The coassembly of midi-metagenomic fractions is therefore not only the most effective but also the most cost-efficient approach compared with current alternatives (Supplemental Tables S6, S7), because sequencing efforts can be distributed across multiple fractions for optimal binning but can still be fully utilized for a combined assembly. The increased sequencing efficiency may be of particular interest for applications of long-read sequencing technologies, which can provide more coherent sequence context at the cost of lower read throughput (Hu et al. 2021). Furthermore, sequencing efforts do not need to be evenly distributed across midi-metagenomic fractions. Thus, the deep sequencing of an unsorted “main” fraction supplemented by auxiliary sorted fractions of lower sequencing depth can further reduce costs in cases in which the research focus lies on the original community composition and the reconstruction of MAGs is only considered a secondary goal.

Importantly, significantly reduced contamination rates are observed in midi-metagenomic MAGs, which is especially noteworthy considering the growing complaints about reference database contaminations caused by insufficiently screened subquality MAGs and SAGs (Breitwieser et al. 2019; Arkhipova 2020; Vollmers et al. 2022).

We could here show that even a simple sorting setup is sufficient for substantial improvements in both the yield and quality of binned MAGs, as long as partial enrichments or depletions of different community members can be achieved. The fractionation of the sampled community can be done using FACS based on many different cell properties, of which the here-utilized cell size and morphology are only the most simple examples (Sturm et al. 2023). In fact, just the act of FACS sorting itself, independent of applied criteria, already represents a general depletion of large multicell aggregates, extracellular DNA, and potential stress susceptible cell types (Wiegand et al. 2021). However, more stringent sorting criteria may further improve the efficiency of the midi-metagenomic approach. Possible criteria could be labeling with fluorescence in situ hybridization (FISH) probes targeting 16S rRNA genes of specific taxonomic groups (Pratscher et al. 2018; Dam et al. 2020) or with function-based enrichments using specific gene or mRNA targeting probes (Kaster and Sobol 2020; Takahashi et al. 2020), radioactive substrate labeling (Lo et al. 2023), fluorescent-labeled antibodies (Müller and Nebe-von-Caron 2010), or sorting based on different autofluorescence spectra caused by species-specific membrane protein compositions (Kang et al. 2020).

However, it needs to be kept in mind that, owing to inherient biases of the sorting process itself, namely, the different enrichment and depletion of specific strains, any definite conclusions on relative abundances within the original community must be based on an unsorted bulk metagenome data set. Such a bulk fraction should therefore generally be included as a reference in the fractionation workflow for each sample (Fig. 2). Because this unsorted bulk metagenome can seamlessly be integrated into the analyses as a dedicated fraction, this does not increase the overall sequencing efforts. Consequently, the total species richness derived by midi-metagenomic assembly can be expected to represent at least the same complexity as a standard bulk metagenome but with the potential addition of strains that are strongly enriched in some of the sorted fractions but sequenced at depths below detection threshold in the bulk metagenome (Figs. 3, 5D; Supplemental Fig. S4; Supplemental Table S8). Consequently, midi-metagenomics may also serve to boost binning efforts in cases in which the variability between samples may turn out not to be sufficient for coabundance-based binning, especially for sample locations that are hard or expensive to access for additional sampling, namely, deep-sea sediments. The exact sorting criteria do not even need to be decided beforehand as a glycerol stock of frozen sample can be revisited for sorting after a preliminary whole-community metagenome analyses.

Although the fractionation process does require access to dedicated equipment such as a FACS- or microfluidic-based cell sorter, which is typically priced at around $230.00 (€200.00), the MDA-free workflow allows for much more streamlined and cost-effective setups compared with mini-metagenomics: Without the highly contamination sensitive MDA-step, the necessity of clean-room standards is removed, greatly simplifying the required infrastructure as well as expertise and potentially even allowing outsourcing the sorting process to external facilities that already possess adequate sorting devices. Consequently, midi-metagenomics represent a novel and improved technique that is widely accessible to the scientific community and can significantly enhance the quality and reliability of prokaryotic genome reconstruction from environmental samples.

## Methods

### Microbial samples

To evaluate midi-metagenomic performance compared with metagenomics, soil samples were collected at the Karlsruhe Institute of Technology (KIT), Campus North, Eggenstein-Leopoldshafen, Germany (49°5′48.8″N, 8°25′55.6″E), during four different periods of time: May 25, 2020, October 7, 2020, August 10, 2021, and February 15, 2022. From each sample, several grams were directly frozen at −80°C immediately after collection for subsequent standard metagenome DNA extraction and sequencing.

Five grams of each sample was then prepared for cell sorting by adding 30 mL of filtered, autoclaved, and UV-sterilized phosphate buffer saline (PBS) solution; brief vortexing to disrupt aggregates and dislocate cells attached to debris; and subsequent pelleting and removal of debris by brief centrifugation at 2000*g*. Sterile glycerol was added to a final concentration of 30% as an antifreezing agent, and the samples were stored at −80°C until further processing. An overview of all samples is given in Table 1.

**Table 1.** Overview of samples and fractions

Sample	Sampling date	Fractions produced
Spring20	May 15, 2020	Only unsorted
Autumn20	October 7, 2020	Unsorted, big & small
Summer21	October 8, 2021	Unsorted, BC, MC, SC, BNC & SNC
Winter21/22	February 15, 2022	Unsorted, BC, MC, SC, BNC & SNC
Autumn22	October 7, 2022	Only unsorted
Winter22/23	March 7, 2023	Unsorted, BC, MC, SC, BNC & SNC

Sample location was at KIT, Campus North, 49°5′48.8′′N, 8°25′55.6′′E. (BC) Big complex, (MC) medium complex, (SC) small complex, (BNC) big noncomplex, (SNC) small noncomplex.

### Fluorescence-activated cell sorting

Prior to sorting, the samples aliquoted for midi-metagenomics were centrifuged for 1 min at 15,871*g* and 20°C. The supernatant was discarded, and after resuspension of the pellet in 1 mL PBS, 5 µL SYBR green I was added to all samples. The samples were then vortexed, incubated for 20 min at 4°C, and subsequently pelleted again by centrifugation for 1 min at 15,871*g*. Each pellet was then washed twice with 1 mL PBS.

Before loading the sample into the FACS (FACSMelody, BD Biosciences), an unlabeled negative control was filtered into a 5 mL FACS tube using a sterile SYSMEX CellTrics filter with a 20 µM mesh size and then diluted with PBS. The negative control was used to compare the difference of fluorescence signals for a correct gating that included only labeled cells. Subsequently, the same procedure was applied to the SYBR-labeled samples. A threshold was set up in order to disregard smaller particles such as debris during the sorting process, and an excitation wavelength of 488 nM was used.

For samples “summer21,” “winter21/22,” and “winter22/23,” cells were sorted into five different groups via gatings based on plotting fluorescence intensity against the forward scatter signal (FSC) and side scatter signal (SSC), which are roughly proportional to cell size and complexity, respectively (Supplemental Table S1; Supplemental Fig. S1). For sample “autumn20,” only two groups were sorted, according to size measured by differences in FSC (Supplemental Table S1). Configurations for fluidic, optical, and electronic settings were kept constant for all sorting runs, as specified in Supplemental Table S1. No compensation was applied, as only one fluorochrome was used. After sorting, the cells were stored at −80°C until further processing. An overview of the fractions produced per sample is included in Table 1.

### DNA extraction

For metagenomics of the unsorted sample, DNA was extracted with the DNeasy PowerSoil kit (Qiagen) following the manufacturer's instructions. For midi-metagenomic community fractions, DNA was extracted directly from FACS-sorted cell suspensions consisting of 4 × 10^6^ cells. First, the cells were freeze-thawed three times using liquid nitrogen and a 60°C water bath. Then, bead beating was performed three times for 30 sec at 6 m/sec using one tube of lysing matrix for each fraction (MP Biomedicals 6914-800) and an FastPrep-24 homogenizer (MP Biomedicals). Beads and cell debris were pelleted by centrifugation at 14,000*g* for 5 min, and the supernatant was subjected to standard alcohol precipitation using 1 volume of 80% isopropanol, 0.1 volume 3 m sodium acetate, and 340 µg linear polyacrylamide. After a subsequent wash step with ice-cold 70% ethanol, the resulting DNA pellet was resuspended with 100 µL PCR-grade water followed by further purification via solid-phase reversible immobilization using 1.5 volume of AMPure XP beads (Beckman Coultier) and final elution in 20 µL 1× TE. All extracted DNA was immediately stored at −20°C until use.

### Polymerase chain reaction for amplicons

Amplicon sequencing was performed using a nested polymerase chain reaction (PCR) approach. Almost full-length PCR products were obtained in a preliminary PCR using 1.25 U OneTaq Quick-Load DNA polymerase (New England BioLabs), 200 µM mixed dNTPs, 500 µM biology-grade bovine serum albumin (BSA; Thermo Fisher Scientific), and 0.2 µM of each universal bacterial forward and reverse primer 27F (5′-AGRGTTYGATYMTGGCTCAG-3′) and 1492R(5′-AGRGTTYGATYMTGGCTCAG-3′). PCR products were purified using DNA Clean & Concentrator-5 columns (Zymo Research) according to the manufacturer's instructions. The purified product was then used as template for a subsequent amplicon PCRs using 0.5 U Q5 high-fidelity DNA polymerase (New England Biolabs), 0.5 U 200 µM dNTP solution mix (New England Biolabs), Q5 high GC enhancer, 0.1 µg/µL BSA (Thermo Fisher Scientific), and 0.2 µM of each universal bacterial primer 341F (5′-AGRGTTYGATYMTGGCTCAG-3′) and 518R (5′-AGRGTTYGATYMTGGCTCAG-3′), targeting the V3 hypervariable region.

### Sequencing

All libraries were prepared using the NEBNext Ultra II FS DNA library prep kit for Illumina (New England Biolabs), according to the manufacturer's instructions. Libraries were sequenced on an Illumina NextSeq 550 (New England Biolabs) device using 300 cycles and a paired-end approach.

### Read processing and assembly

Reads were quality-trimmed and adapter-clipped using Trimmomatic v.0.36, bbduk v.35.69, and cutadapt v.1.14 successively (Martin 2011; Bolger et al. 2014; https://bbmap.org). Overlapping read pairs were identified and merged using FLASH v.1.2.11 (Magoč and Salzberg 2011). For amplicon data sets, reads were clustered into amplicon sequence variants (ASVs) using the Qiime2 pipeline with dada2 (Callahan et al. 2016; Bolyen et al. 2019). To account for different sequencing depths, the produced ASVs were rarefied to a value of 42,000, which was determined via preliminary rarefaction analysis. Rarefied ASVs were subsequently taxonomically classified using RDP Classifier v1.24 (Wang and Cole 2024) and SINA v1.7.2 (Pruesse et al. 2012). Shotgun data sets were arranged into 81 coassembly subgroups representing all possible subsets of three to six data sets per metagenome or midi-metagenome (Supplemental Table S4). To enable standardized assemblies with 15 Gbp total read input each, shotgun data sets were randomly subsampled down to 2.5, 3.75, 3, and 5 Gbp when possible and coassembled at equal amounts for each coassembly subgroup. Additional assemblies with nonuniform sequencing depth distribution were also performed using unsorted metagenome samples as “main” data sets with 12–13 Gbp sequencing depth and 5 “auxilliary” data sets subsampled to 0.4–0.5 Gbp each. Assemblies were performed using MEGAHIT v1.2.9 (Li et al. 2015).

For 16S rRNA gene diversity analysis in shotgun assemblies, 16S rRNA genes were extracted from all assemblies using barrnap (https://github.com/tseemann/barrnap) and clustered at 99% sequence identity level using VSEARCH v2.21.1 (Rognes et al. 2016), and cluster representatives were aligned using SINA v1.7.2. Alignments were then filtered in order to retain only those that fully overlap a defined sequence window of 600 bp roughly representing the hypervariable regions V3–V6. For beta-diversity analyses, OTU tables were generated based the filtered sequences, and read coverages were determined via coverm v0.7.0 (Aroney et al. 2025). Beta-diversity analyses were then performed using Qiime2, and taxonomic classifications were performed as described above.

### MAG reconstruction and analyses

For each coassembly, three different binning tools were used in parallel: Metabat2 v.2.15 (Kang et al. 2019), CONCOCT (Alneberg et al. 2014), and Rosella v.0.4.1 (Newell 2023). For midi-metagenomic approaches, Rosella was substituted for Maxbin (Wu et al. 2016), as Rosella did not function without coabundance information and Maxbin utilizes additional taxonomic and marke-gene criteria that may optimize results for mini-metagenomic and SCG assemblies that lack reliable coverage information (Marine et al. 2014; Kaster and Sobol 2020). Resulting bins were preassessed and filtered using MDMcleaner. Quality categories were then determined based on reassessments using CheckM2 (Chklovski et al. 2023). Taxonomic classifications were based on GTDB-TK v2.1.1 (Chaumeil et al. 2020).

dRep v.3.4.0 (Olm et al. 2017) was employed to identify groups of redundant MAGs created by different assemblies or binning tools and to select the respective most representative MAG. Similarities between MAGs were additionally determined and visualized based on gene content as previously described elsewhere (Howat et al. 2018).

## Data access

All raw sequencing data and processed dereplicated high-quality MAG sequences have been submitted to the NCBI BioProject database (https://www.ncbi.nlm.nih.gov/bioproject/) under accession number PRJNA900514. The complete set of processed sequencing data, including redundant and low-quality MAGs with completeness estimates of at least 50% and contamination estimates <25%, has been submitted to Zenodo (https://doi.org/10.5281/zenodo.13150466).

## Supplemental Material

Supplement 1

Supplement 2
